# The Cure of Deviations of the Nasal Septum

**Published:** 1896-10

**Authors:** Br Vard H. Hulen

**Affiliations:** Galveston, Texas


For the Texas Medical Journal.

### THE CURE OF DEVIATIONS OF THE NASAL SEPTUM.

BR VARD H. HULEN, M. D., GALVESTON, TEXAS.

BY deviations of the septum is understood a lateral curvature of the septum nasi sufficiently marked to interfere with the functions of the anterior nasal cavities. Since Quelmalz first wrote on this subject in 1750 its importance has been recognized, and various means devised and used for its correction. Saws, guages, chisels, knives, drills, trephines, punches, pins, forceps, clamps, tampons, bougies, the galvano-cautery, the ecraseur and compressed air have been used in different ways, but none with entire satisfaction.

It is my wish to bring to the notice of this society an operation that I believe, after a rather extensive observation, to be superior to any method of treatment yet advised. This is the Asch operation, which is a comparatively unknown procedure, although performed first by Dr. Morris J. Asch, of New York, in September, 1883, but a description of the operation was not published until 1890.

During my service as house surgeon of the New York Eye and Eary Infirmary I had the opportunity of studying the operation and its results as performed by its originator, Dr. Asch, as also by Dr. E. Mayer, and other surgeons in this institution.

This operation has given good results in every case where the after-treatment was properly carried out. Knowledge of the technique of the operation and of the after-treatment of the case are of paramount importance, for upon a proper execution of these depends the success or failure of this mode of treatment. The operation being very simple, is easily and quickly performed, and the after-treatment is not dieagreeable or impracticable for any patient.

It is indicated in practically all the deviations of the nasal septum that we are called upon to treat.

It is not the intention of this article to speak of the symptoms, pathology or diagnosis of deflections, but to describe the cure of these troublesome cases.

The special instruments needed in this operation are few; Asch's straight nasal scissors, Mayer's nasal compression forceps and a set of Mayer's nasal tubes. These are the *necessary* instruments; it is convenient to have in addition Asch's small scissors, a pair of Asch's curved scissors, right and left, Asch's gouge and Asch's elevator for use in complicated cases.

The scissors resemble button-hole scissors, one blade three-quarters of an inch long, being lance-shaped and sharp, the other blade having a blunt edge. The forceps are similar to the well-known Adams forceps for refracturing a deflected septum, but the blades of the Mayer forceps, in place of being straight, are set at an obtuse angle with the handle. The set of nasal tubes consists of six vulcanite rubber tubes varying in size from No. 1 to No. 6, No. 1 being the smallest. The tubes are slightly curved, pointed and perforated and oval, conforming to the shape of the nostril.

Before beginning the operation, all the instruments to be used should be sterilized and the nasal cavities thoroughly cleansed and sprayed with antiseptics, for operations on the septum are particularly liable to be followed by sepsis. A ten or twenty per cent solution of cocaine should be applied to the nasal cavities in order to diminish the amount of blood in the parts, for the hemorrhage following the operation is sometimes very profuse. An ice cold Dobell solution in an antomizer should be at hand. It is necessary to put the patient thoroughly under the influence of a general anæsthetic before beginning the operation.

The first step is to break up the synechia and remove the redundant tissue, if any. The scissors are then introduced, the blunt edge on the convex surface and over the point of greatest convexity, and the septum is cut entirely through. The scissors are then withdrawn and again introduced at a right angle to the first position, or as near so as possible, and a second incision made through the deflection. The finger is now introduced into the nose on the convex side and the segments pushed strongly toward the opposite side, care being taken that they are thus thorougly broken. The compression forceps are now introduced and the fractured septum firmly compressed into its proper position. This compression also tends to stop the hemorrhage. The cold Dobell spray is then used and the nostrils cleared of blood clots. A tube of sufficient size to fill the naval cavity is pushed into place on the side of the former occlusion until it just disappears within the nostril. This tube acts as a splint for the broken septum. A smaller tube is then inserted on the other side which keeps it free from blood clots and renders breathing more comfortable, and by making equable pressure prevents subsequent hemorrhages.

The patient is usually confined to bed for forty-eight hours. The after-treatment is extremely important, and should be as follows: During the first twenty-four hours a cold Dobell's solution should be sprayed into the nostril every half hour when the patient is awake; after this less frequently. On the second day the smaller tube is to be removed. On the third day the remaining tube is removed, the nose and tube cleansed and the tube re-introduced. During the following week the tube is to be removed, the nose sprayed and cleansed daily, and the tube reintroduced by the surgeon. The introduction of the tube should not occasion any pain. For a month longer the patient is to be kept under observation, but he is usually able to remove and re-insert the tube daily himself. After this for a week or two the tube should be worn at night only.

The tube is self-retaining, and the removal of it will cause no inconvenience, and is barely noticeable.

Thus at the end of about six weeks it will be found that the patency of the nostril is fully restored, the septum straight and it will remain so permanently.

